# Dual-branch collaborative learning network for crop disease identification

**DOI:** 10.3389/fpls.2023.1117478

**Published:** 2023-02-10

**Authors:** Weidong Zhang, Xuewei Sun, Ling Zhou, Xiwang Xie, Wenyi Zhao, Zheng Liang, Peixian Zhuang

**Affiliations:** ^1^ School of Information Engineering, Henan Institute of Science and Technology, Xinxiang, China; ^2^ School of Information Science and Technology, Dalian Maritime University, Dalian, Liaoning, China; ^3^ School of Artificial Intelligence, Beijing University of Posts and Telecommunications (BUPT), Beijing, China; ^4^ Internet Academy, Anhui University, Hefei, Anhui, China; ^5^ School of Automation and Electrical Engineering, University of Science and Technology Beijing, Beijing, China

**Keywords:** crop disease identification, two-branch collaborative, channel attention, feature cascade, deep learning

## Abstract

Crop diseases seriously affect the quality, yield, and food security of crops. redBesides, traditional manual monitoring methods can no longer meet intelligent agriculture’s efficiency and accuracy requirements. Recently, deep learning methods have been rapidly developed in computer vision. To cope with these issues, we propose a dual-branch collaborative learning network for crop disease identification, called DBCLNet. Concretely, we propose a dual-branch collaborative module using convolutional kernels of different scales to extract global and local features of images, which can effectively utilize both global and local features. Meanwhile, we embed a channel attention mechanism in each branch module to refine the global and local features. Whereafter, we cascade multiple dual-branch collaborative modules to design a feature cascade module, which further learns features at more abstract levels *via* the multi-layer cascade design strategy. Extensive experiments on the Plant Village dataset demonstrated the best classification performance of our DBCLNet method compared to the state-of-the-art methods for the identification of 38 categories of crop diseases. Besides, the Accuracy, Precision, Recall, and F-score of our DBCLNet for the identification of 38 categories of crop diseases are 99.89%, 99.97%, 99.67%, and 99.79%, respectively. 811

## 1 Introduction

Crop diseases have long been one of the most critical factors affecting the stable development of agriculture (Kumari et al., 2019; Chen et al., 2021a; Chamkhi et al., 2022). During the cultivation and growth of crops, if crop diseases are not detected and dealt with promptly, it will miss the best time to control the disease so that the crop diseases cannot be effectively and timely controlled and thus affect the production of crops (Mohanty et al., 2016; Jiang et al., 2022). The annual reduction in food production caused by crop diseases in the world accounts for about one-tenth of the total annual food production. In China, the yearly infestation of crop pests and diseases of different degrees is about 7 billion mu, which directly or indirectly causes the loss of about 85 billion pounds of grain and other economic crops. Meanwhile, the issue is rising yearly, which seriously hinders the stable development of agriculture (Yakhin et al., 2017; Kundu et al., 2021; Darakeh et al., 2022). Countries and regions will benefit from improved ability to predict, detect, negotiate, and effectively address emerging crop disease outbreaks (Carvajal-Yepes et al., 2019; Pandey et al., 2020; Woźniak et al., 2022). As a result, it is vital to design an accurate, efficient, and nondestructive ´ method for crop disease identification for effective disease prevention and precise drug application, which can also recover some economic losses to a large extent.

To cope with the aforementioned issues, many methods have been presented for crop disease identification (Asad, 2022; Fuster-Barceló et al., 2022). Specifically, these existing methods can be categorized as ´ traditional, machine learning, and deep learning methods (Li et al., 2021; Cong et al., 2022a). In the early stages, traditional methods used hand-crafted features for crop disease identification (Dhaka et al., 2021; Yue et al., 2021). Machine learning methods utilize hand-crafted features or semi-automated features to identify crop diseases. Recently, deep learning methods rely on deep network structures to extract features automatically for crop disease identification (Albattah et al., 2022; Kendler et al., 2022). Although most methods based on convolutional neural networks (CNN) have shown superior performance, crop disease images are faced with a wide variety of diseases and irregular distribution of disease spots, so deep learning methods also face challenges.

**Figure 1:**
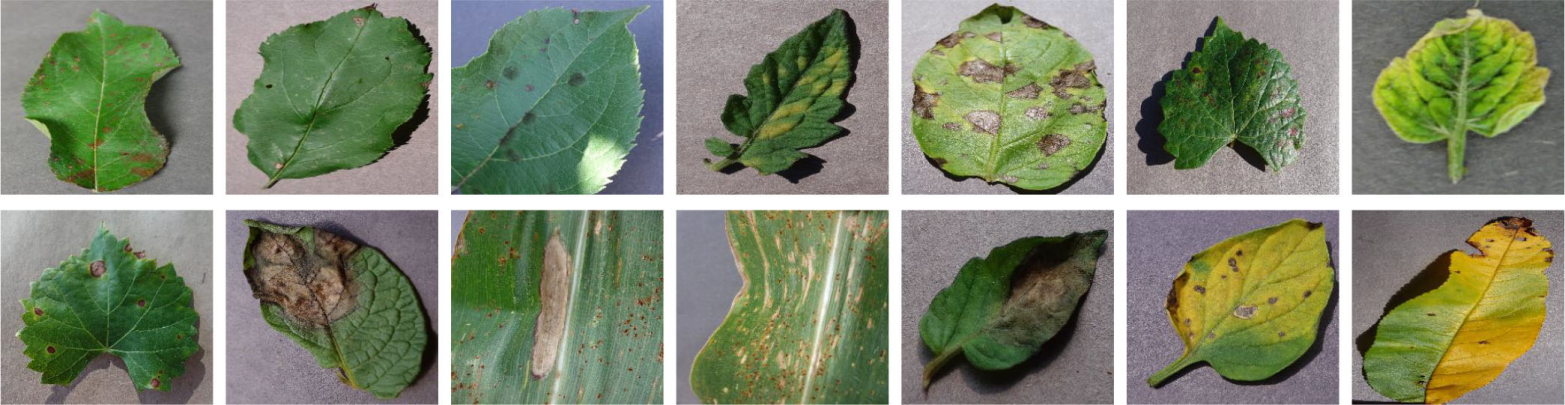
Examples of different crop disease images. These crop disease images are from the PlantVillage data (Hughes et al., 2015), and they face issues of complex lesion types, varying lesion area sizes, and uneven samples.

Currently, most CNN-based methods use small-scale convolutional kernels, and the specialized design utilizes a large number of small-scale convolutional kernels instead of large-scale convolutional kernels to reduce the Flops of the network model to some extent (Viedma et al., 2022; Zhang et al., 2022). Unfortunately, the specialized design may lose some coarse-grained features. In contrast, large convolutional kernels are easy to ignore fine-grained features (Melgar-García et al., 2022; Cong et al., 2022b). **Figure 1** presents some representative examples of different crop disease images, which can clearly observe that these crop disease images face problems such as variable disease types, irregular distribution of disease spots, and varying sizes of disease areas (Cohen et al., 2022). Recently, the advantages of two-branch networks using different learning strategies to integrate different feature information have been widely used in computer vision (Zhang et al., 2021; Xie et al., 2022; Zheng et al., 2022). In contrast, cooperative learning is applied to tracking learning of remote sensing scenes by taking advantage of the synergy and complementarity between different modules (Li et al., 2022b). To sum up, CNN-based methods also face severe challenges in crop disease identification. To take full advantage of coarse-grained, fine-grained, and more abstract level features, we take advantage of the synergistic learning between different modules and the learning strategies of different branches to fully exploit the feature extraction capability of the deep network, we propose a dual-branch collaborative learning network for crop disease identification, called DBCLNet. The network mainly explores the positive effects of collaborative learning strategy, dual-branch module, and feature cascade module on the capacity of crop disease identification. The significant contributions of our proposed DBCLNet model are summarized as follows:

- We propose a dual-branch collaborative module (DBCM), which employs convolutional kernels of different scales to design a dual-branch learning strategy to extract coarse-grained and fine-grained features from crop disease images. Meanwhile, we integrate dual-branch features by drawing on collaborative learning strategies to make our module take advantage of both coarse-grained and fine-grained features.
- We propose a feature cascaded module (FCM) that implements a stacking cascade process by stacking multiple dual-branch collaborative modules, which uses cascading features to enable better utilization of features at a more abstract level and thus improve the discriminatory performance of the DBCLNet model.
- We introduce a focal loss function to address the category imbalance of the samples. Specifically, this loss function decreases the weights of the loss function for categories with a large number of samples. Conversely, the weight of the loss function is increased for the category with a small number of samples. In brief, this strategy effectively reduces the misclassification problem for categories with small samples.

The rest of this paper is organized in detail below. Section 2 provides an overview of work related to crop disease identification methods. Section 3 presents step-by-step details of our proposed DBCLNet model. In Section 4, we present the experimental results and analysis. Section 5 further summarizes the research work and the outlook for future work.

## 2 Related works

Currently, various identification methods are gradually applied to crop disease image identification (Pantazi et al., 2019; Zeng and Li, 2020). We categorize these methods into traditional methods, machine learning methods, and deep learning methods (Flores et al., 2021; Khalifani et al., 2022). In the following, we provide an overview and summary of these research works.

### 2.1 Traditional methods

Utilize digital image processing technology to identify crop disease images *via* preprocessing, hand-made features, feature extraction, and classification (Peña-Barragán et al., 2011). For example, Mondal et al. (Mondal et al., 2017) proposed an entropy-based binarization and naive Bayes classifier method for disease grade prediction of Okra and bitter gourd disease images, which firstly extracted 43 leaf morphological features from these two crops, and then extracted 10 and 9 critical features from the leaf morphological features, respectively. Finally, the predicted results of disease grade were 95% and 82.67%, respectively. However, the accuracy of the method was unsatisfactory due to the limited extraction of valuable features. Huang et al. (Huang et al., 2018) based on the study of powdery mildew and stripe rust faced by winter wheat, they proposed a method to identify wheat lesion images based on Fisher linear discriminant analysis and support vector machine. The technique uses FLDA for feature dimensionality reduction based on selected spectral bands, vegetation indices, and wavelet features, and the classification accuracy of SVM for their identification is 78%. To sum up, the discrimination performance of traditional methods is unsatisfactory because the valuable feature information extracted is limited.

### 2.2 Machine learning methods

Introduce shallow network structures and optimization strategies to semi automatically extract features based on traditional methods, which saves the cost of manually crafting features in the identification process (Feng et al., 2020; Selvaraj et al., 2020). Ma et al. (Ma et al., 2019) designed a crop disease and pest discrimination method based on dual spatiotemporal LandSAT-8 satellite images. It used a synthetic minority oversampling technique to resample the imbalanced training dataset, and the method could achieve 80% crop disease identification accuracy. Chaudhary et al. (Chaudhary et al., 2020). proposed a method based on Ensemble Particle Swarm Optimization, which achieved 96% classification accuracy after 10-fold cross-validation in a recognition classification task for 12 vegetables. Zhang et al. (Zhang et al., 2020b) segmented diseased leaf images using the K-mean clustering algorithm, which extracts the feature vectors of the difference histogram from each segmented defect image based on the intensity values of adjacent pixels and achieves a parity accuracy of 94.4% for the identification of five diseases of cucumber. Li et al. (Li et al., 2020b) proposed shallow CNN with kernel support vector machine and shallow CNN with random forest to discriminate plant diseases, respectively. They have fewer training parameters and higher classification accuracy than traditional CNN. Abdulridha et al. (Abdulridha et al., 2018) significantly improved the detection accuracy of Laurel wilt disease by introducing a multilayer perceptron based on a tree of decisions, which also detected trees infected with Laurel wilt disease at an early stage. Zhang et al. (Weidong et al., 2018) significantly improved the performance of the discriminative model by embedding stacked sparse self-coding into the limitological machine. Khan et al. (Khan et al., 2018) proposed a segmentation method based on correlation coefficients, which first extracted features from selected disease-infected regions using a two-degree pre-training model. Subsequently, they employed a genetic algorithm to choose valuable features. Finally, they used a support vector machine to test the classification accuracy of Lant Village and CASC-IFW up to 98.6%. In general, the machine learning methods are limited by the shallow network, so they capture insufficient feature information. Therefore, the machine learning methods often need to use some feature extraction methods in crop disease identification.

### 2.3 Deep learning methods

Rely on a deep network structure to automatically extract valuable features that drive a nonlinear mapping relationship in crop disease image identification (Zhuang et al., 2022; Li et al., 2022a). For example, Chen *et a*l. (Chen et al., 2020) improved the traditional VGGNet by adding a convolutional layer, swish activation function, and BN layer. In contrast, they were migrating the initialization weights from the pre-trained network on ImageNet, which achieved an average accuracy of 92% on the plant village dataset. Ferentinos et al. (Ferentinos, 2018) designed a new CNN model for crop disease image identification, which experimentally achieved 99.53% classification accuracy on the plant village dataset. Coulibaly et al. (Coulibaly et al., 2019) proposed using transfer learning to solve the problem of CNN’s difficulty in discriminating small samples, and the identification accuracy of this method was 95.00% in Pearl Millet Mildew. Zhang et al. (Zhang et al., 2020a) employed the ranger optimizer to improve the accuracy of EfficientNet for the identification of four diseases of cucumber with 97.00%. Barbedo et al. (Barbedo, 2019) migrated the weights pre-trained on the ImageNet to the GoogLeNet for the PDDB dataset with discrimination accuracy up to 88.00%. Cap et al. (Cap et al., 2022) proposed a LeafGAN with an embedded attention mechanism, which generates disease images from healthy crop images and uses them as training samples to identify the five kinds of cucumber disease images with an accuracy increase of 7.40%. Hu et al. (Cap et al., 2022) proposed a residual neural network model with multidimensional feature compensation, which could discriminate species, coarse-grained diseases, and fine-grained diseases with an accuracy of 85.22% by fusing multidimensional features *via* a compensation strategy. Hu et al. (Hu et al., 2020) proposed a residual neural network model with multidimensional feature compensation, which could discriminate species, coarse-grained diseases, and fine-grained diseases with an accuracy of 85.22% by fusing multidimensional features *via* a compensation strategy. Chen et al. (Chen et al., 2021b) introduced a localization soft attention mechanism based on the pre-trained MobileNet-V2, which embedded localization strategies and migration learning for crop disease images with an accuracy of 99.72%. Haque et al. (Haque et al., 2022) improved Inception-v3 for identifying maize leaf blight, tulip leaf blight, and striped leaf blight, where the best identification result could reach 95.99%. Nandhini et al. (Nandhini et al., 2022) proposed a gated recurrent convolutional neural network to identify crop disease images, in which CNN catches potential features from images in a sequence. Meanwhile, RNN is used to learn temporal features between images in a sequence. Unlike traditional and machine learning methods, deep learning methods only need to design operations such as convolution kernels and pools at different scales to automatically extract contextual information and global and feature information of the images.

## 3 Methodology

**Figure 2:**
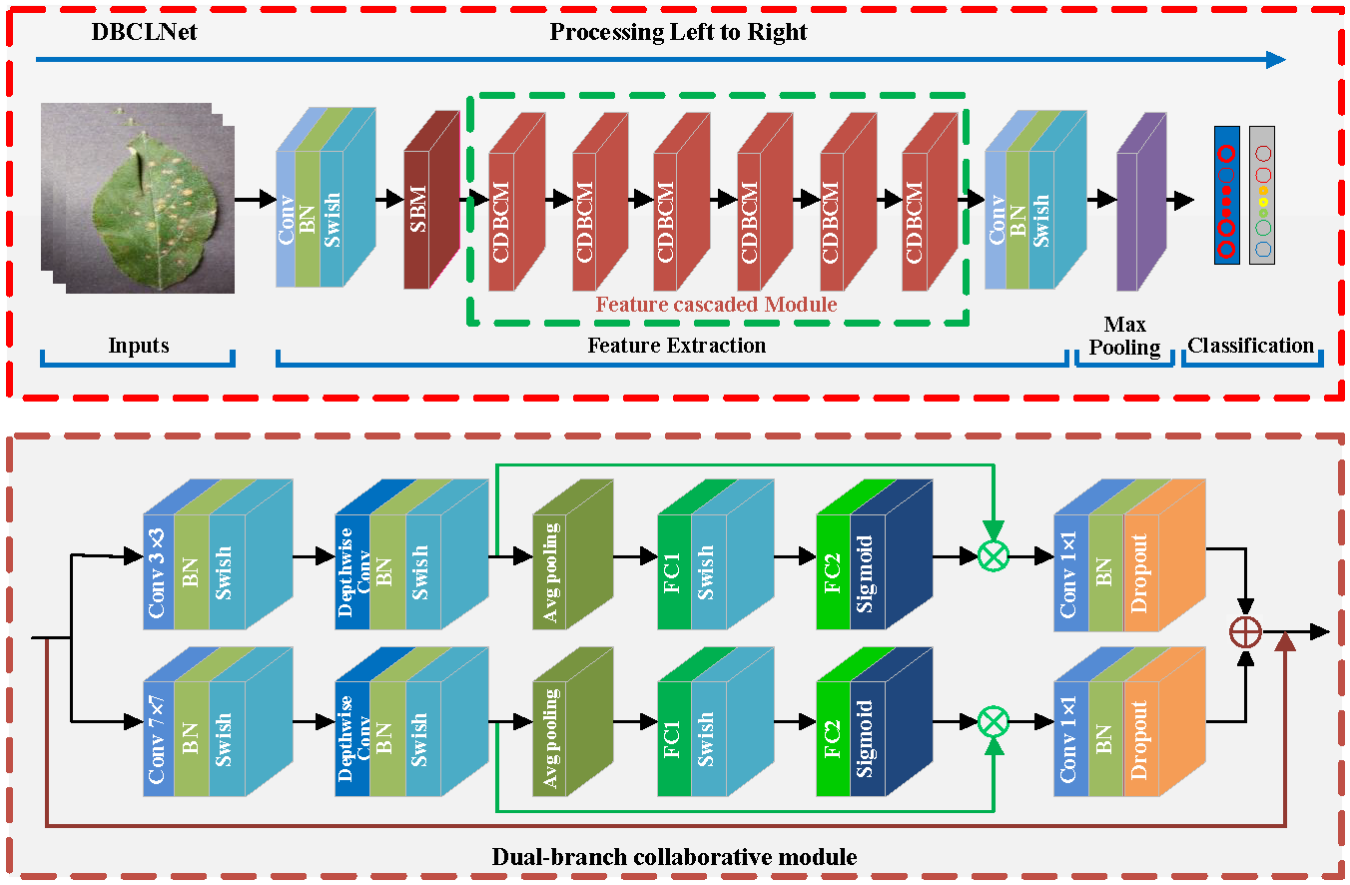
Given a crop disease image of size 224 × 224 × 3 (height × weight × channel), we first expand the number of channels from 3 to 32 dimensions using a convolution kernel of 1 × 1 size. Meanwhile, the base features of the image are extracted, and the size of the feature map is compressed after one single-branch module (SBM). Subsequently, we employ six cascaded DBCMs to form an FCM for coarsegrained and fine-grained feature extraction and integration. The DBCM uses a cooperative learning strategy to integrate features at different levels, and the FCM further extracts features at more abstract levels. Additionally, we add a channel attention mechanism to each branch in the DBCM, and we use maximum pooling for the attention mechanism for branches with smaller convolutional kernels. Similarly, we use average pooling for the branches with larger kernels. Finally, we utilize a 1 × 1 sized convolutional kernel to downscale the number of channels. After maximum global pooling, we flatten the feature matrix into a one-dimensional vector to obtain the classification result by the Softmax function.

Our present the overview architecture of DBCLNet in **Figure 2**. In the input stage, a given crop disease image is transmitted to DBCLNet model after pre-processing. Secondly, we input the preprocessed crop disease images into the Single branch module, which uses the cooperative learning strategy to extract coarse-grained and fine-grained features. Thirdly, we use feature cascaded module to extract more abstract features by stacking and cascading learning strategies. Finally, the feature information is converted into feature vectors in the form of the full connection. Meanwhile, the Softmax function is used to output the classification results in the form of probability.

### 3.1 Network framework

**Figure 2** presents the details of DBCLNet. Our DBCLNet consists of a single-branch module (SBM), a dual-branch collaborative module (DBCM), a feature cascaded module (FCM), and a fully connected module. SBM is designed to extract the basic features of crop disease images, DBCM is employed to extract coarse-grained and fine-grained features of crop disease images, FCM is utilized to extract features at the more abstract level of crop disease images, and FCM is used for the category probability output of the final classification results. In addition, **Table 1** reports the details of each module in the DCBLNet model.

**Table 1:** Details of each module of DCBLNet.

Layers (type)	Input Size	Output Size	Repeat	#Parammater
Input_1	3 × 224 × 224	3 × 224 × 224	1	0
Conv_1	3 × 224 × 224	32 × 224 × 224	1	658
SBM_1	32 × 224 × 224	16 × 112 × 112	1	1024
DBCM_1	16 × 112 × 112	24 × 112 × 112	2	11868
DBCM_2	24 × 112 × 112	40 × 56 × 56	3	33114
DBCM_3	40 × 56 × 56	80 × 28 × 28	4	172484
DBCM_4	80 × 28 × 28	112 × 14 × 14	4	385636
DBCM_5	112 × 14 × 14	192 × 14 × 14	3	1438708
DBCM_6	192 × 14 × 14	320 × 7 × 7	2	509234
Conv_2	320 × 7 × 7	1280 × 7 × 7	1	292634
MaxPooling_1	192 × 14 × 14	320 × 7 × 7	1	0
Flatten_1	1280	1280	1	0
Linear_1	1280	38	1	909512

Total Trainable Parameters: 3,754,872

### 3.2 Dual-branch collaborative module

Inspired by the feature extraction capacity of convolutional kernels of different scales (Li et al., 2020a; Lian et al., 2021; Chen et al., 2022), we design a dual-branch collaborative module (DBCM) by taking advantage of the collaborative complementarity of convolutional kernels of different scales for feature extraction. The module is called the dual-branch cooperative module. It is worth noting that our designed module includes shallow feature extraction, deep feature extraction, channel attention, and collaborative learning. In the following, we present the design details of DBCM step by step.

#### 3.2.1 Shallow feature extraction

CNN is a classic representative of deep neural networks inspired by biological neural networks (Dong et al., 2022). The network structure of CNN is different from other deep learning models, which employ local connections instead of full connections to extract contextual feature information of the images (Kong et al., 2021). Additionally, CNN utilizes shared weights instead of assigning weights to each input to reduce the number of parameters. Based on these advantages, CNN has better generalization performance in the field of computer vision.

Inspired *via* weighted feature fusion of CNN (Dong et al., 2022), we employ thirty-two 1 × 1 convolution kernel to perform feature image up-dimensional mapping on the input image. Specifically, we use any of the convolution kernels to convolve the red, green, and blue channels of the input image and integrate them into one feature map until the thirty-two feature maps are solved. We mainly utilize multiple convolution kernels to reconstruct multiple feature maps so that the feature information of the input image can be used by the dual-branch collaborative module as much as possible. Concretely, the initial convolution of the input image X*_h,w,c_* is defined as:


(1) $$X_{h,w,n}^{f}=\sigma \left(\sum_{i,j,c}\left(X_{h,w,c}K_{i,j,c,n}+Bias_{i,j,c,n}\right)\right),$$

where *h*, *w*, and *c* represent the height, width, and channel of the input image, respectively. **K** *_i,j,c,n_* denotes the *n^th^* convolution kernel of the input image in the *i^th^* row and *j^th^* column of the *c^th^* channel, and *n* denotes the number of convolution kernels. **Bias** *_i,j,c,n_* denotes the bias value of the convolution operation, *σ*(·) represents the Swish activation function of the convolution operation, and ${\text{X}}_{h,w,n}^{f}$ denotes the *n^th^* feature map of the output. Swish = *x* · Sigmoid(*βx*), *β* represents a constant or trainable parameter. In addition, the Swish activation function is upper bound-free and lower bound-free, smooth, and non-monotonic. Meanwhile, the Swish outperforms ReLU on deep models. Subsequently, we redefine the integration of shallow feature information as $\;X_{S}^{c}\in {\mathbb{R}}^{h,w,c}$ . The shallow feature information includes both a large amount of valuable feature information and a large amount of useless feature information. We use a dual-branch network in the deep feature extraction stage to extract useful and remove useless features. Our work defines the shallow feature extraction process as a single-branch module. The feature information we extract in the initial stage is used as the input for the deep feature extraction stage.

#### 3.2.2 Deep feature extraction

In depth feature extraction stage, we propose extracting coarse-grained and fine-grained features using convolutional kernels of different scales for the input features, in which the coarse-grained mainly includes thetexture and global feature information of the images, and the fine-grained feature mainly consists of the detail and local feature information of the images. Subsequently, we define the dual-branch convolution process as:


(2) $$X_{c1}^{c}=\text{Swish }\left({\text{CNN }}_{3\times 3}\left(X_{S}^{c}\right)+B_{1}^{c}\right),$$

(3) $$X_{c2}^{c}=\text{Swish }\left({\text{CNN }}_{7\times 7}\left(X_{S}^{c}\right)+B_{2}^{c}\right),$$

where CNN_3×3_ and CNN_7×7_ denote the 3×3 and 7×7 convolution operation in the upper and lower branches of the deep feature extraction stage, **B**_1_ and **B**_2_ represent the bias vales in the upper and lower branches of the deep feature extraction stage, and $X_{c1}^{c}$ and $X_{c2}^{c}$ denote the fine-grained and coarse-grained features in the upper and lower branches of the deep feature extraction stage. Despite the fact that we can capture the coarse-grained and fine-grained features of the image better in the step, the network model parameters are complex and inefficient. To reduce the parameters of the model and improve the efficiency of the network, we introduced a depthwise convolution operation in the subsequent stage of the initial feature extraction of the DBCM. Subsequently, we could redefine the features of the dual-branch as follows:


(4) $$X_{d1}^{c}=\text{Swish }\left(D{\text{epthwise CNN}}_{3\times 3}\left(X_{c1}^{c}\right)+B_{1}^{c}\right),$$

(5) $$X_{d2}^{c}=\text{Swish }\left(D{\text{epthwiseCNN }}_{3\times 3}\left(X_{c2}^{c}\right)+B_{2}^{c}\right),$$

where $X_{c1}^{c}$ and $X_{c2}^{c}$ are the features obtained from Eqs. (??0) and (3). $X_{d1}^{c}$ and $X_{d2}^{c}$ are the features after depthwise convolution. The convolution operation can not only reduce the model’s parameters and improve the model’s efficiency but also capture the local features of the channel dimension. How to fully use the feature information of different channel levels is the problem we solve later.

#### 3.2.3 Channel attention

**Figure 3:**
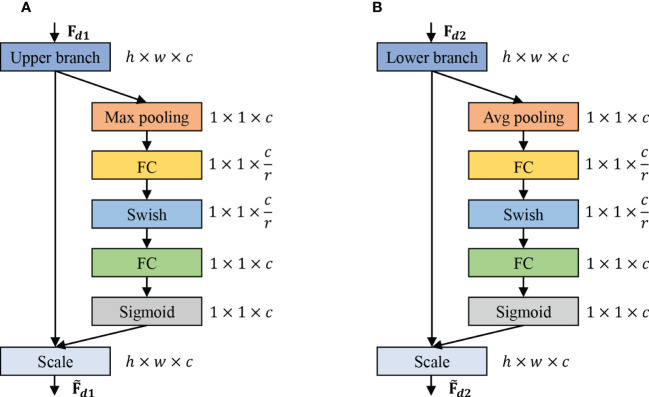
The schema of the Max pooling and Avg pooling operations. **(A)** Channel attention mechanism of the upper branch, which is used to refine fine-grained features. **(B)** Channel attention mechanism of the lower branch, which is used to refine coarse-grained features.


We introduce the channel attention module to exploit the features of different channel levels further. Meanwhile, we introduce the maximum pooling channel attention for the upper branch and average pooling channel attentionfor the lower branch. **Figure 3** reports the flowchart of max pooling (Maxpooling) and average pooling (Avgpooling). The channel attention includes global information embedding and adaptive calibration. We first consider the interdependence between each channel in the output features for the global information embedding of the upper and lower branches. For the upper branch, we utilize the maximum pooling to retain more image texture information, which also reduces the model parameters to a certain extent and thus prevents the network from overfitting. Mathematically, the maximum pooling can be expressed as:

(6) $$F_{\text{max}}^{c}=\text{MaxPooling }\left(X_{d1}^{c}\right)=\sum_{{\text{Ω}}_{i,j}^{c}\in {\mathbb{R}}_{h,w,c}}\underset{\left(p,q\right)\in {\text{Ω}}_{i,j}^{c}}{\max\;}x_{d1}^{c}(i,jt),$$

where $F_{\text{max}}^{c}$ denotes the matrix that integrates the maximum pooled values of all rectangular regions Ω associated with the *c^th^* feature map. $x_{d1}^{c}\left(i,j\right)$ denotes the element located at (*p,q*) in the rectangular region Ω of the *c^th^* feature map. For the lower branch, we utilize average pooling for retaining as much background feature information of the image as possible. Mathematically, the average pooling can be defined as:


(7) $$F_{\text{avg}}^{c}=\text{Avg Pooling }\left(X_{d2}^{c}\right)=\frac{1}{\left|{\text{Ω}}_{i,j}^{c}\right|}\sum_{{\text{Ω}}_{i,j}^{c}\in {\mathbb{R}}_{h,w,c}}x_{d2}^{c}\left(i,j\right),$$

where $F_{\text{avg}}^{c}$ denotes the matrix that integrates the average pooled values of all rectangular regions Ω associated with the *c^th^* feature map. $\left|{\text{Ω}}_{i,j}^{c}\right|$ indicates the number of elements in the rectangular area ${\text{Ω}}_{i,j}^{c}$ . To take advantage of the aggregation feature in the squeeze operation of the upper and lower branches, we perform the operation after it to capture the channel-related dependencies. Subsequently, the adaptive recalibration process ofthe upper and lower branches is defined as follows:


(8) $$F_{s1}=F_{ar}\left(F_{\text{max}},W\right)=\text{Sigmoid }\left(\text{Swish }\left(W_{1}F_{\text{max}}\right)W_{2}\right),$$

(9) $$F_{s2}=F_{ar}\left(F_{\text{avg}},W\right)=\text{Sigmoid }\left(\text{Swish }\left(W_{1}F_{\text{avg}}\right)W_{2}\right),$$

where $W_{1}\in {\mathbb{R}}^{\frac{C}{r}\times C}$ and $W_{2}\in {\mathbb{R}}^{C\times \frac{C}{r}}$ . To limit the model’s complexity and benefit generalization, we parameterize the gating mechanism by forming a bottleneck of two fully connected layers around the nonlinearity, i.e., a reduced-dimensional decay rate of *r*.


(10) $${\tilde{F}}_{d1}^{c}=F_{\text{scale}}\left(F_{\text{d}1}^{c},F_{\text{s}1}^{c}\right)=F_{\text{s}1}^{c}F_{\text{d}1}^{c},$$

(11) $${\tilde{F}}_{d2}^{c}=F_{\text{scale}}\left(F_{\text{d}2}^{c},F_{\text{s}2}^{c}\right)=F_{\text{s}2}^{c}F_{\text{d}2}^{c},$$

where ${\tilde{F}}_{d1}=\left[{\tilde{F}}_{d1}^{1},{\tilde{F}}_{d1}^{2},\ldots ,{\tilde{F}}_{d1}^{C}\right]$ , ${\tilde{F}}_{d2}=\left[{\tilde{F}}_{d2}^{1},{\tilde{F}}_{d2}^{2},\ldots ,{\tilde{F}}_{d2}^{C}\right]$ . $F_{\text{scale}}\left(F_{\text{d}1}^{c},F_{\text{s}1}^{c}\right)$ and $F_{\text{scale}}\left(F_{\text{d}2}^{c},F_{\text{s}2}^{c}\right)$ based on the channel-wise multiplication betweenthe scaler $F_{\text{s}1}^{c}$ and $F_{\text{s}2}^{c}$ , as well as the feature map $F_{\text{d}1}^{c}\in {\mathbb{R}}^{h\times w}$ and $F_{\text{d}2}^{c}\in {\mathbb{R}}^{h\times w}$ , respectively. We fuse them in the subsequent stages to empower our DBCM to consider the complementary information of the advantageous features of the upper and lower branches.

#### 3.2.4 Collaborative learning

To fully take into account the complementary advantages of the features of our DBCM integration of the upper and lower branches. The upper branch focuses on capturing fine-grained feature information, and the branch focuses on capturing coarse-grained feature information. Therefore, the process of integrating coarse-grained and fine-grained features is called collaborative learning. To fully exploit the low-level features, we integrate the input features into the coarse-grained and fine-grained feature levels in the feature integration process. Eventually, the process of collaborative learning of these features is defined as:


(12) $$F_{\text{DBCM}}^{c}=\text{Concatenate }\left(X_{s}^{c},F_{\text{d}1}^{c},F_{\text{d}2}^{c}\right),$$

where $X_{s}^{c}$ is the base feature extracted from Eq. (1). $F_{\text{d}1}^{c}$ is extracted step by step from the upper branch *via* Eqs. (2), (4), (6), (8), and (10). $F_{\overset{\circ }{m}d2}^{c}$ is extracted step by step from the lower branch *via* Eqs. (3), (5), (7), (9), and (11). $F_{\text{DBCM}}^{c}$ denotes the final extracted features *via* the DBCM. Thanks to our design, we usedeep convolution to capture deep feature information in the feature extraction process, and use different scales and different strategies of convolution to capture features with different advantages. In addition, we added the channel attention mechanism to DBCM (Tan and Le, 2019), which contains a max pooling layer or an average pooling layer with two fully connected layers. Similar to the traditional attention mechanism, the channel attention mechanism acts on the feature map from the perspective of the channel, which makes the network pay more attention to the disease spots on the leaves and reduce the weight of the disease-free regions better to capture the disease spot features in the leaves. To comprehensively consider the features at a more abstract level and the lost feature by convolution, we then designed a cascaded stacked DBCM module called the feature stacked module.

### 3.3 Feature cascaded module

redInspired by the MBConv network [60], we cascade multiple DBCMs for extracting features at a more abstract level, called the feature cascaded module. Meanwhile, the DBCM mentioned above is the basic unit module that constitutes the FCM. An DBCM unit can be defined as a function of **F**_FCM_ = DBCMM(**F**_BDCM_), where DBCM is the dual-branch collaborative module, **F**_FCM_ is output feature, **F**_DBCM_ is input feature with $F_{\text{DBCM}}\in {\mathbb{R}}^{h,w,c}$ , where *h* and *w* are the hight and width of the feature map, and *c* is the number of channels. Subsequently, an FCM can be represented by a series of DBCM combinations, and the stack-cascaded process is defined as:


(13) $$\begin{array}{ll} F_{\text{FCM}} & ={\text{DBCM }}_{1}\left(F_{\text{DBCM}}\right)⊙\cdot \cdot \cdot ⊙{\text{DBCM }}_{s}\left(F_{\text{DBCM}}\right)\\ =\underset{i=1\ldots s}{⊙}{\text{DBCM }}_{i}^{{\text{Iter}}_{i}}\left(F_{\text{DBCM}}\right). \end{array},$$

where ${\text{DBCM}}_{i}^{{\text{Iter}}_{i}}$ represents the DBCM is represented Iter*_i_* times in stage *i*. In our FCM, we designed to repeatedly stack 6 DBCM. Specifically, each DBCM was repeated 2,3,4,4,3 and 2 times, that is to say, the repetition times of DBCM on both sides were reduced, and the repetition times of DBCM in the middle were more. The unique design makes it difficult for our network to lose key feature information in deep feature extraction. Then, the feature map obtained by FCM is reduced by 1×1 convolution. Finally, we obtain the final discrimination result through maximum pooling, and full connection layer.

### 3.4 Loss function

Most current classification studies focus on the cross-entropy loss function in traditional classification tasks (Bahri et al., 2020). Most current classification studies focus on the cross-entropy loss function in traditional classification tasks. Specifically, the process constructs a probability distribution between the true and predicted values while it uses a cross-entropy loss function to describe the distance between these two probability distributions. It minimizes the cross-entropy loss by iterative training to obtain the optimal training model. Subsequently, the cross-loss function for binary classification is defined as follows:


(14) $$\begin{array}{ll} {\text{Loss}}_{\text{ce}} & =-y_{t}\text{log}\left(y_{p}\right)-\left(1-y_{t}\right)\text{log}\left(1-{\text{y}}_{\text{p}}\right)\\ =\{\begin{array}{l} -\log\;\left(y_{p}\right),{\text{y}}_{\text{t}}=1\\ -\log\;\left(1-y_{p}\right),{\text{y}}_{\text{t}}=0 \end{array} \end{array},$$

where *y_t_* represents the true value, *y_p_* represents the predicted value, *y_t_* = 1 denotes the predicted result is a positive sample, and *y_t_* = 0 denotes the predicted results is a negative sample. *y_p_* is the result of the activation function output in the range [0,1]. Note that the more positive sample with higher output probability, the smaller the loss. In contrast, the more negative samples with a smaller output probability, the smaller the loss. In general, the effectiveness of the cross-entropy loss function for the multiclassification discrimination problem appears unsatisfactory.

**Figure 4:**
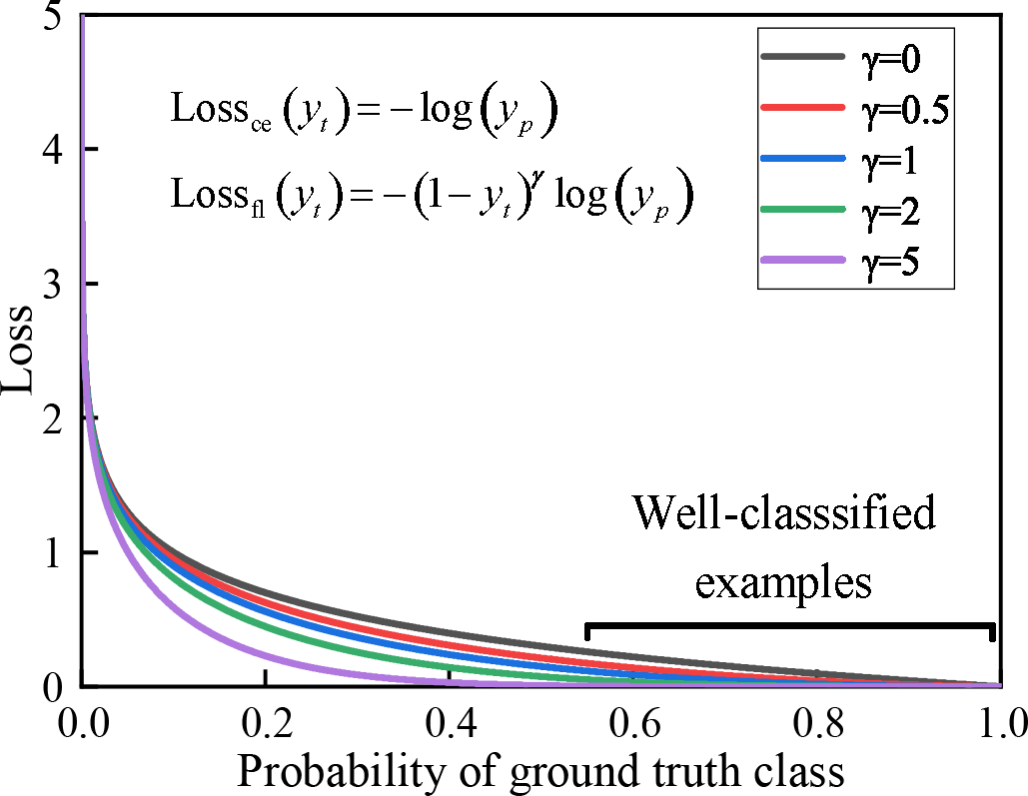
The effect of the focal loss function on the relationship between the true class and the loss function. When γ > 0, the discriminative model focuses on difficult and misidentified samples as the loss continues to decrease.

Since the Plant village dataset is faced with category imbalance, that is to say, the number of samples varies significantly between different crop images. These issues can also bring challenges to crop disease identification. For example, similar features are repeatedly extracted for the same crop during feature extraction, resulting in higher classification accuracy for categories with a more significant number of samples and lower classification accuracy for categories with fewer samples. Therefore, we employ a focal loss function superior to the cross-entropy loss function (Bahri et al., 2020). It weakens the problem of sample imbalance by strengthening the categories with few samples and weakening the categories with many samples. Its expression is defined as:


(15) $${\text{Loss}}_{\text{fl}}=\{\begin{array}{l} -\alpha {\left(1-y_{p}\right)}^{\gamma }\log\;y_{p},y_{t}=1\\ -\left(1-\alpha \right)y_{p}^{\gamma }\log\;\left(1-y_{p}\right),y_{t}=0 \end{array},$$

where *y_t_* and *y_p_* are defined as shown in Eq. (14). *α* is the equalization factor, which is used to equalize the number of samples from different categories. *γ* is the adjustment factor, which is utilized to adjust the decayrate of the different category sample weights. In a real classification task, this function decreases the weight of loss for samples with higher prediction probability and increases the weight of loss for samples with lower prediction probability. This strategy makes our discriminative model more focused on the sample imbalance problem. As shown in **Figure 4**, it shows a loss in terms of dynamically scaled cross-entropy, where the scaling factor *γ* decreases to zero as the confidence level of the correct category increases. Extensive statistical results show that our model has the best discriminatory performance when *α* = 2 and *γ* = 0.25.

### 3.5 Experimental data

**Figure 5:**
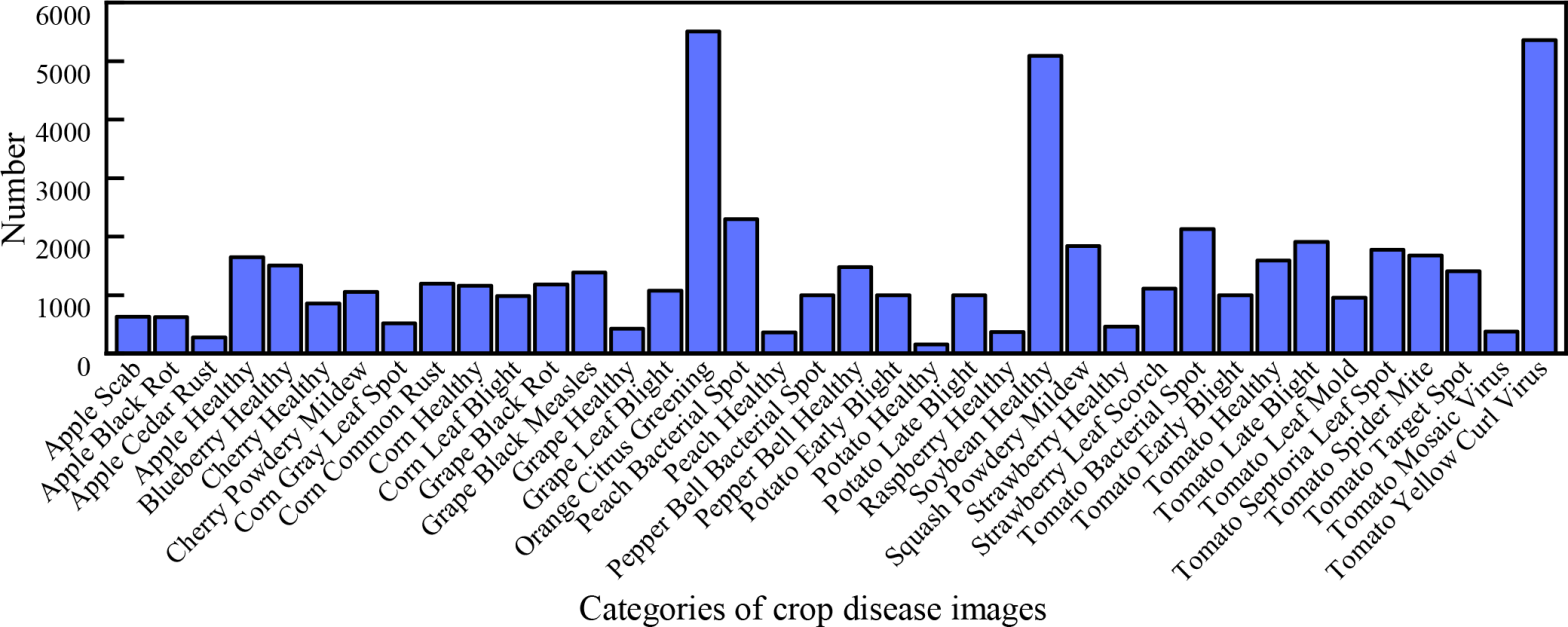
Histogram of the different categories of samples in the PlantVillage data (Hughes et al., 2015). From left to right are the 630 apple scab images, 621 apple black rot images, 275 apple cedar rust images, 1645 apple healthy images, 1502 blueberry healthy images, 854 cherry healthy images, 1052 cherry powdery mildew images, 513 corn gray leaf spot images, 1192 corn common rust images, 1162 corn healthy images, 985 corn leaf blight images, 1180 grape black rot images, 1383 grape black measles images, 423 grape healthy images, 1076 grape leaf blight images, 5507 orange citrus greening images, 2297 peach bacterial spot images, 360 peach healthy images, 997 pepper bell bacterial spot images, 1477 pepper bell healthy images, 1000 potato early blight images, 152 potato healthy images, 1000 potato late blight images, 371 raspberry healthy images, 5090 soybean healthy images, 1835 squash powdery mildew images, 456 strawberry healthy images, 1109 strawberry leaf scorch images, 2127 tomato bacterial spot images, 1000 tomato early blight images, 1591 tomato healthy images, 1909 tomato late blight images, 952 tomato leaf mold images, 1771 tomato septoria leaf spot images, 1676 tomato splider mite images, 1404 tomato target spot images, 373 tomato mosaic virus images, and 5357 tomato yellow curl virus images, respectively.

**Figure 6:**
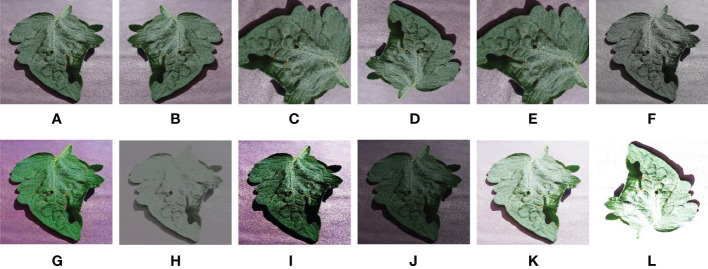
Example representation of an enhanced sample. From left to right are **(A)** raw image, **(B)** mirror rotated image, **(C)** rotated 90 degree image, **(D)** rotated 180 degree image, **(E)** rotated 270 degree image, **(F)** low-saturated image, **(G)** high-saturated image, **(H)** low-contrasted image, **(I)** high-contrasted image, **(J)** low-brightened image, **(K)** high-brightened image, and **(L)** Overexposed image.

Our DBCLNet with 12 compared methods is tested on the PlantVillage data (Hughes et al., 2015). Specifically, this publicly available dataset has a total of 54304 images of crop leaves, mainly including 38 healthy and diseased images of 14 types of crops, including apples, blueberries, cherries, potatoes, tomatoes, etc. **Figure 5** shows the histogram distribution of the number of samples in different categories of the PlantVillage dataset. From **Figure 5**, we can observe that the samples in the dataset are incredibly uneven. The unbalanced samples face severe challenges in the discriminative and generalization performance of the model. To balance the number of samples from different categories to improve the generalization performance of our model,we adopt the strategy of data augmentation. Specifically, we utilize mirror flip, rotation, and contrast change strategies to enhance the data for the categories with fewer samples. As shown in **Figure 6**, we show a typical example of crop image augmentation before and after. Notably, the augmented PlantVillage data has a total of 87867 samples. Meanwhile, the samples of different categories are better balanced after data augmentation.

## 4 Experimental results

In this section, we mainly introduce the experimental settings, evaluation matrices, identification evaluation, and ablation study. To fairly and comprehensively evaluate the discriminatory performance of our method, our DBCLNet is compared with twelve deep learning methods, including traditional network models: AlexNet (Krizhevsky et al., 2017), VGGNet (Simonyan and Zisserman, 2014), and GoogLeNet (Szegedy et al., 2015); low-weight network models: MobileNet (Howard et al., 2017) and ShuffleNet (Zhang et al., 2018); deep network models: ResNet50 (He et al., 2016), DenseNet1 (Huang et al., 2017), and DenseNet2 (Too et al., 2019); attention network models: EfficientNet (Tan and Le, 2019), RegNet (Radosavovic et al., 2020), ViT (Dosovitskiy et al., 2020), and CoAtNet (Dai et al., 2021). We utilize the recommended parameter settings to run the source codes provided by the authors to obtain the best results from different methods.

### 4.1 Experimental settings

We method run on a Windows 10 PC with AMD Ryzen 5 3600X Central Processing Unit (CPU) at 3.80 GHz, 32-GB memory, NVIDIA GeForce GTX 1080Ti GPU, and Pytorch deep learning framework.

In our NBCLNet, we set the batch size is 16, the optimizer is AdamW with *β*_1_ = 0.9 and *β*_1_ = 0.999 optimizer decay rates. Meanwhile, the wight decay is 0.05. Our DBCLNet are trained with 50 iterations, with the base learning rate is set as 10^-3^. Additionally, the learning rate schedule is cosine decay, and the label smooth is 0.1. According to our DBCLNet input requirements all crop disease images are set to a size of 224×224×3. The number of samples for the original category imbalance was increased to 87867 data sets with the number of balanced samples by data augmentation. Subsequently, we build samples according to the ratio of 8:1:1 for training set, validation set and test set. The training set is used to train and optimize our DBCLNet model, the validation set is used to verify the validity of our model, and the test set is used to test the discrimination performance of our model.

### 4.2 Evaluation matrices

In evaluation matrices, we selected accuracy (*A*_Acc_), precision (*A*_Pre_), recall (*A*_Rec_), and F1 score (*A*_Fscore_) as evaluation metrics for agricultural disease image identification. For each classification result, theymay be categorized into four cases: true positive (TP), false negative (FN), false positive (FP), and true negative (TN). *A*_Acc_ indicates the ratio of the total number of correctly predicted samples to the total number of tested samples, and the higher accuracy indicates the better discrimination performance of the proposed method. *A*_Pre_ represents the proportion of true samples among all predicted positive samples, while a higher value indicates a better discriminative performance of themethod. *A*_Rec_ indicates that accurate prediction is true in the proportion of all true, and the higher the value, the better the discrimination performance of this method. *A*_Fscore_ is the combined average of accuracy *A*_Acc_ and recall *A*_Rec_, and its higher value indicates the better identification performance of the method. In addition, **Table 2** reports the details of the expressions for each matrices.

**Table 2:** Error matrix for accuracy verification of of the identification results of crop disease images.

Item		Reference information		Row total	Evaluation metrics
		True	False		
Identification results	True	TP	FN	TP+FN	A_Rec_ = TP/(TP+FN)
False	FP	TN	FP+TN	–
Columns total		TP+FP	FN+TN	*N*	–
Evaluation metrics		A_Pre_ = TP/(TP+FP)	A_Acc_ = 1 - *A_R_*	–	$A_{\text{Fscore}}=2\times \frac{A_{Pre}\times A_{Rec}}{A_{Pre}+A_{Rec}}$

### 4.3 Identification evaluation

To demonstrate the effectiveness and generalization performance of our DBCLNet using the PlantVillage data comparing 12 deep learning methods. Meanwhile, our DBCLNet and the compared method are configured according to the same training, test, and validation set. We chose the traditional network models (Simonyan and Zisserman, 2014; Szegedy et al., 2015; Krizhevsky et al., 2017), low-weight network models (Howard et al., 2017; Zhang et al., 2018), deep network models (He et al., 2016; Huang et al., 2017; Too et al., 2019), and attention network models (Tan and Le, 2019; Dosovitskiy et al., 2020; Radosavovic et al., 2020; Dai et al., 2021) to compare our methods fairly and comprehensively. In addition, the source code and running parameters of all the compared methods are provided by the authors.

**Table 3** exhibitions the identification results of 38 crop disease images tested by different methods. **For traditional network models**, AlexNet (Krizhevsky et al., 2017) obtained the lowest classification accuracy *A*_Acc_, precision *A*_Pre_, recall *A*_Rec_, and F1 score *A*_Fscore_. Because of its simple and shallow network structure, AlexNet (Krizhevsky et al., 2017) has poor performance in multi-classification of crop disease images. VGGNet (Simonyan and Zisserman, 2014) and GoogLeNet (Szegedy et al., 2015) increase the depth of the network making them better than AlexNet (Krizhevsky et al., 2017) in feature extraction, but their classification performance is also unsatisfactory due to the limitation of the influential network model. In general, the traditional network models are limited by the depth and effective network structure, which makes them difficult to solve the multi-classification problem of crop disease images.

**Table 3:** Identification results of different deep learning methods tested on the PlantVillage dataset for 38 crop disease images.

Method	A_Acc_ ↑	*A*_Prm_ ↑	*A*_Rec_ ↑	*A*_Fscore_ ↑
AlexNet (Krizhevsky et al., 2017)	0.8261	0.9038	0.8609	0.8789
VGGNet (Simonyan and Zisserman, 2014)	0.9139	0.9332	0.8879	0.8978
GoogLeNet (Szegedy et al., 2015)	0.9284	0.9499	0.8874	0.8949
MobileNet (Howard et al., 2017)	0.9483	0.9633	0.9171	0.9201
ShuffleNet (Zhang et al., 2018)	0.9467	0.9372	0.9532	0.9551
ResNet50 (He et al., 2016)	0.9569	0.9578	0.9560	0.9570
DenseNet1 (Huang et al., 2017)	0.9713	0.9665	0.9749	0.9748
DenseNet2 (Too et al., 2019)	0.9975	00.9968	0.9951	0.9948
EfficientNet (Tan and Le, 2019)	0.9913	0.9939	0.9887	0.9913
RegNet (Radosavovic et al., 2020)	0.9884	0.9904	0.9851	0.9845
ViT (Dosovitskiy et al., 2020)	0.9879	0.9909	0.9842	0.9863
CoAtNet (Dai et al., 2021)	0.9927	0.9933	0.9919	0.9915
DBCLNet	0.9989	0.9997	0.9967	0.9979

Optimal: red Suboptimal: blue.

For low-weight network models, MobileNet (Howard et al., 2017) introduces depth-separable convolution to build lightweight deep neural networks, while it introduces a width and a resolution multiplier to effectively trade-off between latency and accuracy. Therefore, their discrimination performance for crop disease images is better than VGGNet (Simonyan and Zisserman, 2014), and GoogLeNet (Szegedy et al., 2015) thanks to their effective network structure. ShuffleNet (Zhang et al., 2018) introduces pointwise group convolution and channel shuffle for neural networks to save computational resources, which significantly reduces the computational overhead while retaining the accuracy of the model. Therefore, ShuffleNet (Zhang et al., 2018) has the discrimination ability similar to that of MobileNet (Howard et al., 2017) for crop disease image discrimination. Overall, the low-weight network models have a more efficient structure than the traditional network models. Therefore, they have better discrimination performance than VGGNet (Simonyan and Zisserman, 2014), and GoogLeNet (Szegedy et al., 2015). However, their classification accuracy is also somewhat insufficient due to the restriction of network depth.

**For deep network models**, ResNet50 (He et al., 2016) introduces both deep network structure and residual mechanism making it have better feature extraction ability and convergence speed. Therefore, ResNet50 (He et al., 2016) is better than traditional networks and low-weight network models for crop disease image identification. DenseNet1 (Huang et al., 2017) introduces a skip dense connectivity module and a deep network layer based on ResNet50 (He et al., 2016) to make its discrimination ability better than that of ResNet50 (He et al., 2016). DenseNet2 (Too et al., 2019) explores the discrimination ability of different deep learning methods for crop disease images, while further optimizing DenseNet1 (Huang et al., 2017) significantly improves the discrimination performance of DenseNet2 (Too et al., 2019).In general, the deep network model improves the discrimination performance of the network model at the expense of network depth and computational resources.

**For attention network models**, EfficientNet (Tan and Le, 2019) employs a strategy with channel attention mechanism stacking to make the model have better feature extraction capability, so it performs better in identifying crop disease images. RegNet (Radosavovic et al., 2020) proposes that the adopted design space design strategy follows an incremental design approach, which has a better discriminatory performance. ViT (Dosovitskiy et al., 2020) uses a transformer relying on the number of samples of training data being large enough and the image content being rich sufficient for image classification, which has achieved better identification results in the identification of crop disease images. CoAtNet (Dai et al., 2021) effectively combines convolutional neural network and transformer, and at the same time embedding attention into the model, CoAtNet (Dai et al., 2021) achieves better discrimination results than ViT (Dosovitskiy et al., 2020) for crop disease image discrimination. Overall, the attention model has the advantages of effective network structure, deep feature extraction layer, and attention mechanism. It is worth noting that although DenseNet2 (Too et al., 2019), EfficientNet (Tan and Le, 2019), and CoAtNet (Dai et al., 2021) achieved better discrimination results for crop disease image identification, they are still lower than our DBCLNet. Thanks to our design, our DBCLNet can better extract coarse-grained, fine grained, and more abstract-level features of images. Hence, our network model has better discriminative performance than the compared methods.

As **Table 4** shows the Flops, training time, parameters and memory of different discriminatory models. Compared to most methods, our DBCLNet has a significant advantage in terms of training time. Although our DBCLNet is worse than ShuffleNet in terms of Flops, Parameters, and Memory, our DBCLNet still has some advantages over other methods. In general, our method not only has high discriminative performance but also outperforms most methods in model complexity.

**Table 4:** Complexity analysis of different identification models.

Method	Flops ↓	Training time ↓	Parameters ↓	Memory ↓
AlexNet (Krizhevsky et al., 2017)	312.11 M	3.3 h	15.6 MB	2.77 MB
VGGNet (Simonyan and Zisserman, 2014)	7.63 G	2.5 h	126.7 MB	62.59 MB
GoogLeNet (Szegedy et al., 2015)	1.59 G	18.7 h	6.7 MB	30.03 MB
MobileNet (Howard et al., 2017)	227.71 M	3.1 h	24.4 MB	50.39 MB
ShuffleNet (Zhang et al., 2018)	150.6 M	2.8 h	2.2 MB	20.85 MB
ResNet50 (He et al., 2016)	8.22 G	3.4 h	24.4 MB	109.69 MB
DenseNet1 (Huang et al., 2017)	2.88 G	15.5 h	7.6 MB	147.10 MB
DenseNet2 (Too et al., 2019)	3.02 G	15.7 h	7.8 MB	152.25 MB
EfficientNet (Tan and Le, 2019)	399.3 M	4.0 h	5.0 MB	79.40 MB
RegNet (Radosavovic et al., 2020)	203.75 M	4.7 h	2.6 MB	23.53 MB
ViT (Dosovitskiy et al., 2020)	6.72 G	49.3 h	20.5 MB	339.01 MB
CoAtNet (Dai et al., 2021)	4.15 G	30.5 h	13.0 MB	231.11 MB
DBCLNet	275.51 M	2.8 h	3.6 MB	57.17 MB

Optimal: red; Suboptimal: blue.

**Figure 7:**
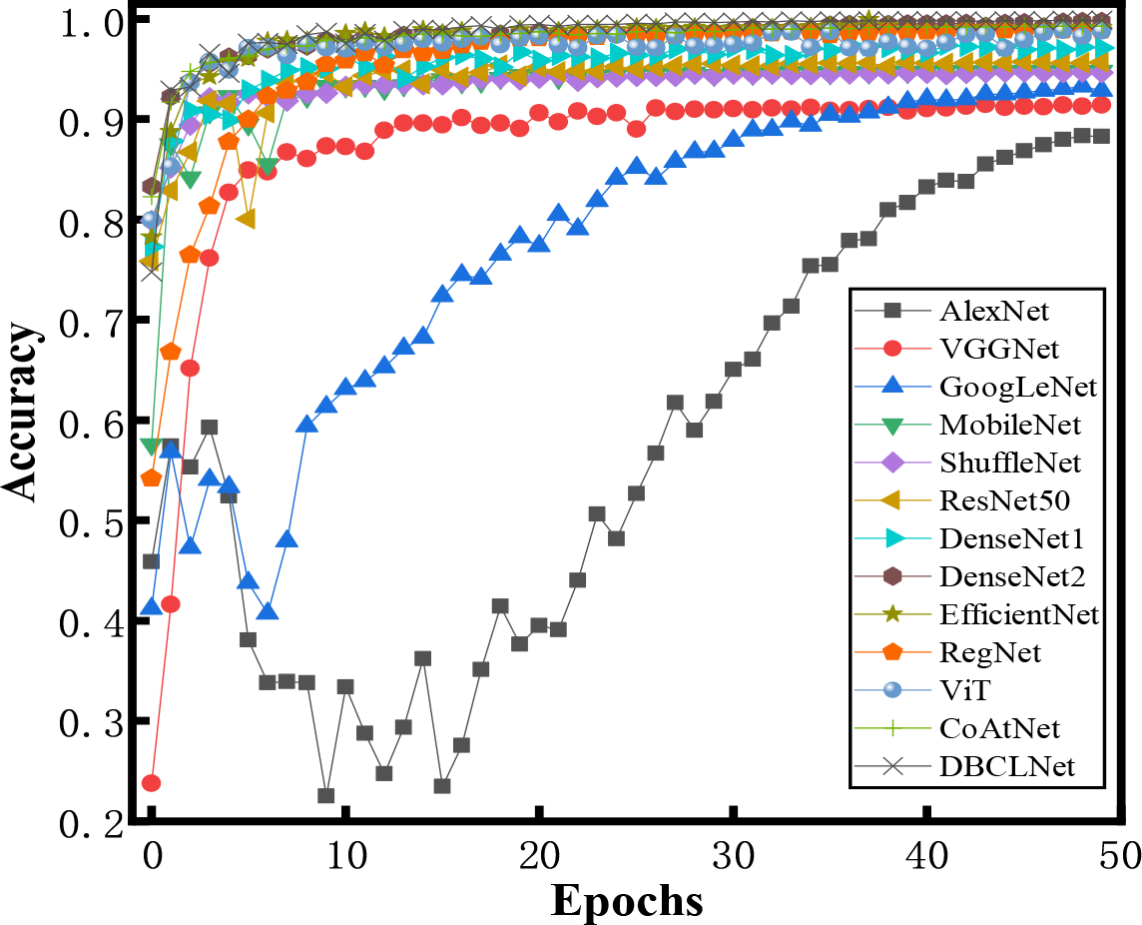
Histogram of the different categories of samples in the augmented PlantVillage data (Hughes et al., 2015).

**Figure 7** shows the accuracy of different methods for crop disease image identification under different iterations. For traditional network models, the accuracy of AlexNet (Krizhevsky et al., 2017) and GoogLeNet (Szegedy et al., 2015) does not increase significantly with the increase in the number of iterations. They tend to be stable when the number of iterations is around 35. VGGNet (Simonyan and Zisserman, 2014), RegNet (Radosavovic et al., 2020), and MobileNet (Howard et al., 2017) do not have ideal identification accuracy with a small number of iterations. MobileNet (Howard et al., 2017), ResNet50 (He et al., 2016), EfficientNet (Tan and Le, 2019), and ViT (Dosovitskiy et al., 2020) are still able to obtain good discrimination with fewer iterations. With the increase in the number of iterations, our DBCLNet rapidly increases to the highest classification accuracy and tends to be stable at about 10 iterations.

**Figure 8:**
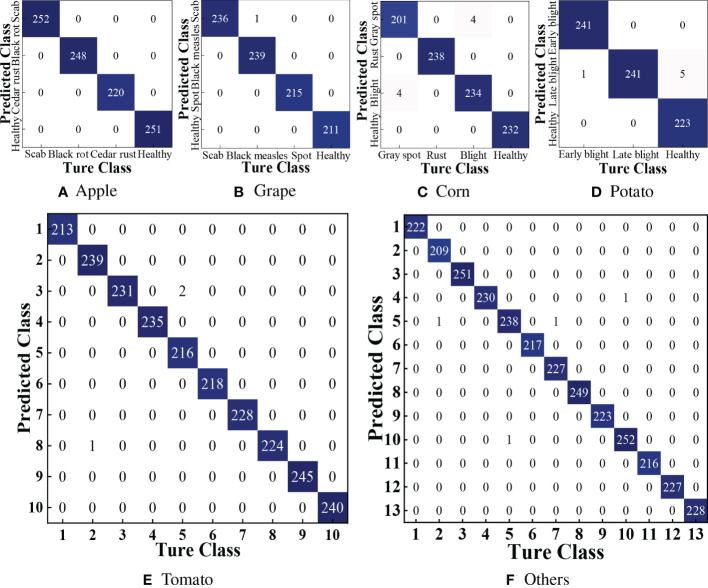
Confusion matrix of our DBCLNet is tested on the 38 crop disease images. **(A)** Confusion matrix of apple, **(B)** Confusion matrix of grape, **(C)** Confusion matrix of corn, **(D)** Confusion matrix of Potato, **(E)** Confusion matrix of Tomato, and **(F)** Confusion matrix of others.

From **Figure 8**, we can observe the confusion matrix plot of DBCLNet for the test samples. We can clearly observe that DBCLNet can achieve more than 99.00% identification results for most crop disease images. It is worth noting that the identification result of our DBCLNet for the apple disease images is 100.00%, while its discrimination result for the Grape disease image was only wrong by one.

### 4.4 Ablation study

To explore the positive impact of each module in our DBCLNet on discriminatory performance, we performed the following ablation study on the augmented PlantVillage data (Hughes et al., 2015), including (1) our DBCLNet without batch standard normalization layer (-w/o BSNL), (2) our DBCLNet without single-branch module (-w/o SBM), (3) our DBCLNet without dual-branch collaborative module (-w/o DBCL), (4) our DBCLNet without feature cascaded module (-w/o FCM).

As shown in **Table 5**, the following discriminatory results can be observed: (1) -w/o BSNL has less effect on the identification results, it doesn’t have feature extraction so it has less impact. (2) -w/o SBM is employed to extract the underlying features so it has less impact on the discriminatory performance. (3) -w/o DBCL has the greatest impact on the identification results, it focuses on extracting coarse-grained and fine-grained features, so it significantly impacts the discrimination results. (4) -w/o FCM has a greater impact on the identification results, it focuses on a more abstract level of extraction and therefore has a greater impact on the discrimination results. Our full model has the best results for the identification of cropdisease images. From **Table 5**, we designed each module to impact our DBCLNet positively. Our full model has the highest *A*_Acc_, *A*_Prm_, *A*_Rec_, and *A*_Fscore_ scores. Overall, our DBCLNet can obtain optimal discrimination performance thanks to the special design of each module.

**Table 5:** Discriminatory results of different modules for the implementation of ablation studies on test samples.

Method	A_Acc_ ↑	*A*_Prm_ ↑	*A*_Rec_ ↑	*A*_Fscore_ ↑
-w/o BSNL	0.9477	0.9360	0.9552	0.9569
-w/o SBM	0.9723	0.9758	0.9745	0.9765
-w/o DBC	0.8771	0.8885	0.8677	0.8859
-w/o FCM	0.8994	0.9046	0.8951	0.9067
DBCLNet (full model)	0.9989	0.9997	0.9967	0.9979

Optimal: red; Suboptimal: blue.

## 5 Discussion

This paper presented a dual-branch collaborative learning network for crop disease identification. We first provide a comprehensive overview of the current research in the crop disease image identification field. Meanwhile, we also summarize the advantages and disadvantages of various methods and the wide application of deep learning methods in this field. Subsequently, we explained the proposed DBCLNet in detail. Our DBCLNet comprises a single-branch module, a dual-branch collaborative module, and a cascaded feature module. The SBM extracts basic features of crop disease images, and the DBCM focuses on extracting coarse-grained. Fine-grained features from crop disease images and the FCM mainly extract crop disease image features at a more abstract level. Extensive experiments on the augmented PlantVillage data demonstrate that my DBCLNet has good discrimination ability for 38 types of crop disease images.

Despite the satisfactory results of my DBCLNet for the crop disease image identification issue, our method has some limitations. On the one hand, our method is inferior to other crops in disease identification of corn and potato because the disease characteristics of corn and potato are challenging to extract. However, our method outperforms other comparative methods for identifying these two crops. On the other hand, our method uses a deep network structure to extract coarse-grained, fine-grained, and more abstract features, improving discrimination performance at algorithm complexity’s cost. Compared with the low-weight network model, our method has a more complex network structure and a more significant number of parameters. We future will focus our research on two issues: extracting fine features and optimizing network models.

## Data availability statement

The original contributions presented in the study are included in the article/Supplementary Material. Further inquiries can be directed to the corresponding author.

## Author contributions

WeiZ and XS conceived and designed the experiments. WeiZ and LZ performed most of experiments. LZ and XX analyzed the data. WeiZ, XS, and WenZ wrote the manuscript. ZL and PZ provided the technical support. All author contributed to the article and approved the submitted version.
